# Protein Spatial Structure Meets Artificial Intelligence: Revolutionizing Drug Synergy–Antagonism in Precision Medicine

**DOI:** 10.1002/advs.202507764

**Published:** 2025-08-07

**Authors:** Anqi Lin, Chang Che, Aimin Jiang, Chang Qi, Antonino Glaviano, Zhijie Zhao, Zhirou Zhang, Zaoqu Liu, Ziyao Zhou, Quan Cheng, Shuofeng Yuan, Peng Luo

**Affiliations:** ^1^ Donghai County People's Hospital (Affiliated Kangda College of Nanjing Medical University); Department of Oncology Zhujiang Hospital, Southern Medical University Lianyungang 222000 China; ^2^ Xinling College Nantong University Nantong Jiangsu 226000 China; ^3^ Department of Urology Changhai Hospital Naval Medical University (Second Military Medical University) Shanghai 200443 China; ^4^ Institute of Logic and Computation TU Wien Wien 1040 Austria; ^5^ Department of Biological Chemical and Pharmaceutical Sciences and Technologies University of Palermo Palermo 90123 Italy; ^6^ Department of Plastic and Reconstructive Surgery Shanghai Ninth People's Hospital Shanghai JiaoTong University School of Medicine Shanghai 200011 China; ^7^ Department of Oncology Zhujiang Hospital Southern Medical University Guangzhou China; ^8^ Institute of Basic Medical Sciences Chinese Academy of Medical Sciences and Peking Union Medical College Beijing 100730 China; ^9^ College of Veterinary Medicine Sichuan Agricultural University Chengdu 611130 China; ^10^ Department of Neurosurgery Xiangya Hospital Central South University Changsha Hunan 410008 China; ^11^ National Clinical Research Center for Geriatric Disorders Xiangya Hospital Central South University Changsha 410008 China; ^12^ Department of Infectious Disease and Microbiology The University of Hong Kong‐Shenzhen Hospital Shenzhen 518009 China; ^13^ Department of Microbiology State Key Laboratory of Emerging Infectious Diseases Carol Yu Centre for Infection School of Clinical Medicine Li Ka Shing Faculty of Medicine The University of Hong Kong Hong Kong China

**Keywords:** artificial intelligence, drug antagonism, drug synergy, protein spatial structure

## Abstract

Targeted drug design and development, as a core area of modern pharmaceutical research, critically depends on the assessment of protein site druggability as a fundamental component. This review systematically examines the latest research progress and application prospects of drug synergy and antagonism prediction methods that integrate protein three‐dimensional spatial structure with artificial intelligence (AI) technologies. This review showcases the molecular biological mechanisms of drug synergism vs antagonism mediated by transcription factors, signal pathway regulation, and membrane transport proteins, and subsequently delves into the molecular structural basis of protein–drug interactions, including precise identification methods for drug binding sites, optimization strategies for molecular docking techniques, and the mechanisms and structural characteristics of multi‐target drugs. The review systematically evaluates the practical application progress of AI technologies, especially machine learning and deep learning algorithms, in predicting drug synergy–antagonism effects, as well as the methodological approaches for constructing and evaluating the performance of AI prediction models that integrate multi‐source biological data. These research findings provide a solid theoretical foundation for the precision treatment of cancer, infectious diseases, and metabolic disorders, with significant clinical and translational implications for advancing personalized medicine strategies in clinical practice and facilitating the rational design and development of novel multi‐target drugs.

## Graphical abstract

This review comprehensively examines the applications of protein three‐dimensional structures and artificial intelligence in investigating drug synergistic and antagonistic effects. The research findings provide compelling evidence for the precision treatment of cancer and metabolic diseases, thereby promoting the development of personalized medicine and multi‐target drug design strategies.

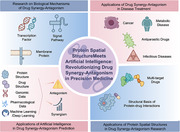

## 1 Introduction

Drug combinations serve as important tools for studying biological systems and revealing relationships between different cellular processes,^[^ ^1^, ^2^, ^3^, ^4^ ^]^ playing a critical role in the investigation of drug synergistic and antagonistic effects.^[^ ^5^ ^]^ Synergism occurs when the therapeutic efficacy of two or more drugs used in combination exceeds the additive effect or expected outcome of lower‐order interactions when used individually, while antagonism refers to drug interactions that result in decreased therapeutic effects of the individual agents. Multi‐drug combination therapeutic strategies, particularly in cancer treatment, have become established approaches in clinical practice,^[^ ^6^, ^7^, ^8^ ^]^ with combinations of chemotherapeutic agents representing prominent examples of this strategy.^[^ ^9^ ^]^ Clinically, drug synergism is generally preferred due to its ability to enhance therapeutic effects, particularly in improving efficacy against infected hosts.^[^ ^10^ ^]^ Protein spatial structure plays a crucial role in elucidating drug action mechanisms, particularly in target selection and the prediction of drug interactions.^[^ ^11^, ^12^ ^]^

The three‐dimensional spatial structure of proteins determines the specificity of drug binding to their targets, including key factors such as the size, shape, and polarity of binding sites; only when these specificity factors are complementary can drugs effectively exert their intended effects.^[^ ^13^, ^14^ ^]^ Notably, protein spatial structures exhibit dynamic characteristics, with different structural conformations leading to configurational changes in binding sites, thereby significantly affecting drug affinity.^[^ ^15^ ^]^ Among these binding sites, protein active sites that perform key functions are typically composed of specific amino acid residues, and their spatial arrangement and relative positions are crucial for the specific binding process between drugs and targets.^[^ ^12^, ^16^ ^]^ Additionally, the global conformation and local secondary structure of proteins can also significantly impact the specificity of drug binding sites, thereby potentially inducing drug synergistic or antagonistic effects. In recent years, through the application of artificial intelligence technologies, especially machine learning and deep learning algorithms, researchers have been able to extract key molecular features from vast amounts of experimental data, systematically identify synergistic or antagonistic relationships between drug combinations, and facilitate the discovery and development of new drug combinations.^[^ ^17^, ^18^, ^19^ ^]^

Meanwhile, protein spatial structure prediction technologies provide atomic‐level, high‐resolution three‐dimensional protein structure models, thereby revealing precise binding modes and interaction mechanisms between targets and drug molecules.^[^ ^20^, ^21^ ^]^ AI technologies systematically analyze massive experimental data at cellular and molecular levels, accurately predicting therapeutic responses of drug combinations under various pathological conditions based on multi‐level biological information, thus optimizing the design and application strategies of drug combinations.^[^ ^22^ ^]^ When integrated with high‐resolution protein spatial structure data, advanced artificial intelligence algorithms provide more accurate targeting predictions in drug discovery and development, significantly enhancing the prediction accuracy of drug combination synergistic effects and safety profiles.^[^ ^23^, ^24^ ^]^

The dynamic properties of proteins significantly impact drug interaction mechanisms. Proteins in various conformational states exhibit marked differences in their interaction effects with drugs.^[^ ^25^, ^26^ ^]^ As conventional methods struggle to accurately predict protein conformational changes, predicting drug combination effects becomes increasingly complex. Compared to single‐target approaches, multi‐target drugs precisely regulate multiple key disease aspects, not only enhancing therapeutic efficacy but also addressing drug resistance issues and reducing adverse effects, thus demonstrating broad application potential and significant clinical value.^[^ ^22^ ^]^ However, multi‐target drugs face application challenges due to complex mechanisms of action, specifically: i) drug–target interactions may simultaneously exhibit both antagonistic and synergistic effects; ii) while achieving complementary therapeutic effects through multiple pathway regulation, these drugs may trigger potential adverse effects due to regulatory imbalances; iii) complex pharmacokinetic profiles may affect absorption, distribution, metabolism, and excretion processes, significantly impacting drug efficacy.^[^ ^27^, ^28^, ^29^ ^]^ The integration of artificial intelligence with protein spatial structure analysis plays a crucial role in drug development. By predicting synergistic–antagonistic mechanisms of drug combinations through artificial intelligence, researchers can optimize combination strategies while elucidating molecular mechanisms of drug action, providing precise theoretical guidance for drug development.

This paper systematically reviews research progress in integrating protein three‐dimensional spatial structure with artificial intelligence for drug synergy and antagonism prediction. In multi‐target drug design, dynamic conformational changes of target proteins decisively influence drug molecule binding modes, significantly affecting therapeutic outcomes. Protein structures function as network information carriers that, when analyzed with artificial intelligence technologies—particularly machine learning and deep learning algorithms—enable the extraction of specific features from complex drug–target interaction networks, facilitating high‐precision prediction of drug synergistic or antagonistic effects. AI technologies show promise in screening and optimizing drug combinations by analyzing molecular structural characteristics and target interaction patterns, though current approaches face limitations in generalizability and interpretability that must be addressed for reliable clinical translation. These technologies can also accurately predict potential side effects, thereby minimizing the occurrence of adverse drug reactions. Multi‐target drugs can simultaneously act on multiple pathogenic targets, offering significant therapeutic prospects in clinical applications; however, they also face key challenges regarding pharmacokinetic parameter complexity and precise target selection. Artificial intelligence technologies can effectively address these challenges, systematically facilitate the optimization of drug combination regimens, and significantly enhance the clinical efficacy and safety of multi‐target drugs. To meet the clinical needs of personalized medicine, artificial intelligence technologies can integrate and analyze patients' genomic data and phenotypic information to precisely identify individualized clinical responses to drug synergistic effects, thereby facilitating the development and implementation of personalized treatment plans. The integration of high‐throughput screening technologies with artificial intelligence methodologies and protein three‐dimensional structural information represents a multidisciplinary approach that is expected to significantly accelerate future new drug development, systematically optimize treatment regimens, and provide more precise theoretical guidance and reliable clinical support for multi‐drug combination therapies in the era of precision medicine.

## 2 Research on Biological Mechanisms of Drug Synergy–Antagonism

**Figure 1:**
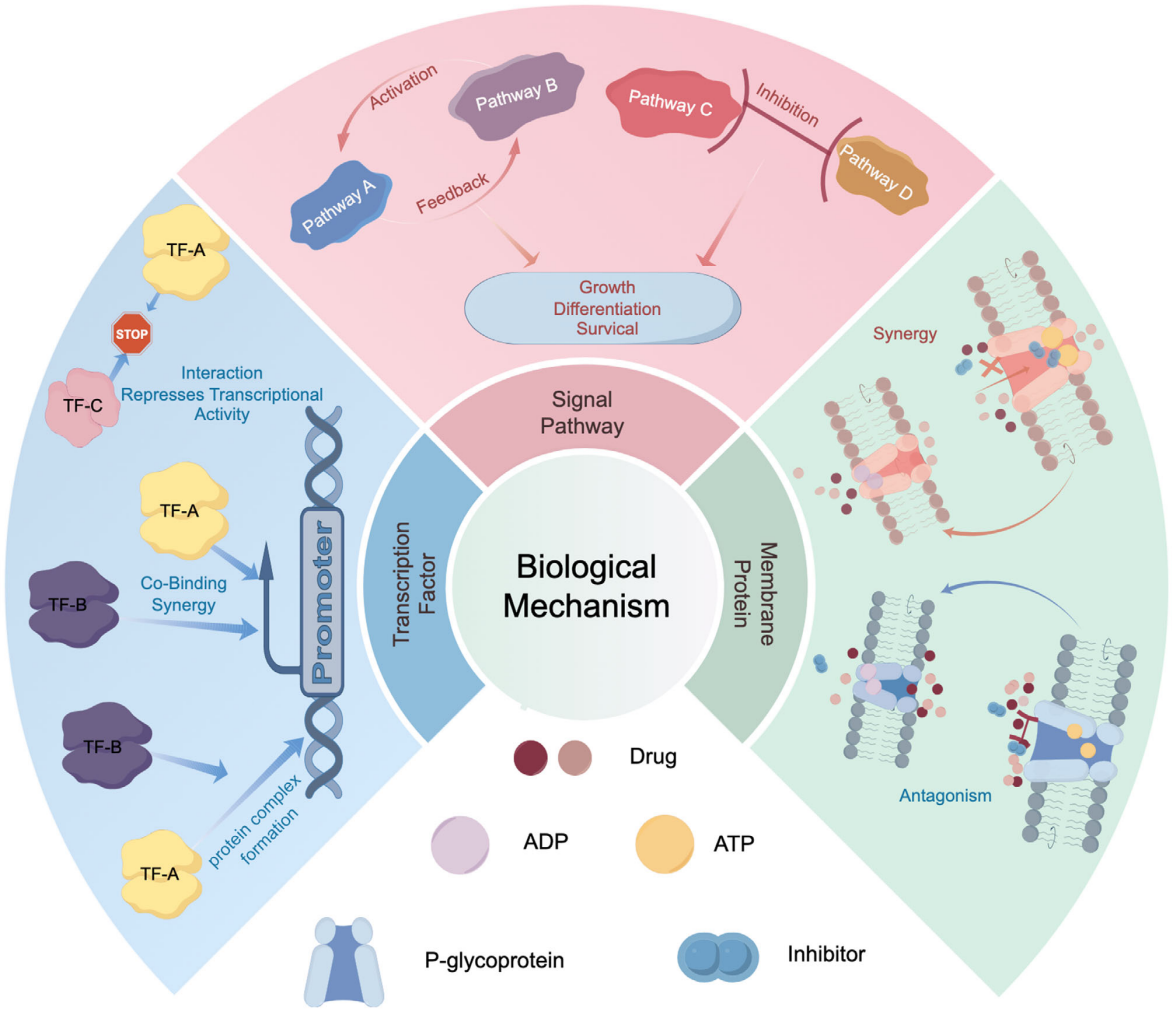
Fundamental biological mechanisms of drug synergy and antagonism. This figure systematically illustrates the fundamental biological mechanisms of drug synergy and antagonism, including: 1) Transcription factors: Transcription factors interact with each other to form functional protein complexes that specifically bind to promoters. Multiple transcription factors synergistically bind to he promoter region of the same gene, thereby forming efficient transcription initiation complexes. Specific transcription factors competitively bind to the same regulatory sequences in DNA. 2) Signaling pathways: Activation of pathway B by signaling pathway A is significantly enhanced through synergistic effects and signaling pathway A can amplify its own effect through positive feedback mechanisms; Some pathways exhibit antagonistic effects (possibly by inhibiting key regulatory components of other pathways). 3) Membrane transport proteins: When one drug effectively inhibits the transport function of P‐glycoprotein, the intracellular concentration otf other drugs increases accordingly, thereby inducing synergistic effects. When multiple drugs competitively bind to P‐glycoprotein, they lead to significantly reduced intracellular drug concentrations, resulting in antagonistic effects. This figure was generated using the Figdraw‐based tool platform.

The biological mechanisms underlying drug synergy–antagonism operate through multi‐level molecular regulatory networks, including transcription factors, signaling pathways, and membrane transport proteins (**Figure** **1**). Transcription factors exert synergistic or antagonistic effects through coordinated activation or competitive binding to specific target genes. Signaling pathway interactions can amplify or inhibit drug effects, while negative feedback mechanisms or cross‐inhibition pathways can lead to reduced therapeutic efficacy. P‐glycoprotein, a key membrane transport protein, facilitates drug efflux from cells, thus reducing intracellular concentrations and therapeutic outcomes. Conversely, inhibiting P‐glycoprotein can promote drug accumulation and enhance treatment efficacy.

### 2.1 Transcription Factor‐Mediated Synergy–Antagonism

The interaction mechanisms between transcription factors constitute a key molecular basis through which drugs exert synergistic or antagonistic effects. Different transcription factors can precisely regulate gene expression networks through direct mutual binding or the formation of multi‐protein complexes, thereby significantly enhancing synergistic effects.^[^ ^30^, ^31^ ^]^ Two or more transcription factors can synergistically bind to the promoter region of the same gene, thereby forming transcription initiation complexes and orchestrating gene expression programs.^[^ ^32^ ^]^ Additionally, specific transcription factors may inhibit each other's transcriptional activity through competitive binding to identical DNA recognition sequences or through protein–protein interactions, thus producing antagonistic effects at the molecular level.^[^ ^33^ ^]^

Peng et al. discovered that the transcription factor EHF forms a functional complex with co‐activator AJUBA in gastroesophageal adenocarcinoma (GEA) cells, thereby synergistically promoting the activation of the KRAS signaling pathway. The co‐localization pattern of EHF and AJUBA in GEA cell nuclei forms a highly stable transcriptional regulatory complex that significantly alters the higher‐order chromatin structure of target genes, induces chromatin open states in specific enhancer regions, promotes the recruitment and binding of transcriptional activation complexes, and thereby upregulates multiple oncogenes associated with tumor progression. The molecular interaction between EHF and AJUBA exhibits significant time‐dependent characteristics in GEA cells, which dynamically regulates the chromatin accessibility of target genes and precisely influences the spatiotemporal expression patterns of genes associated with the KRAS signal transduction pathway. This molecular‐level synergistic regulatory mechanism results in controlled open‐closed transition patterns of chromatin conformation across different time phases, thereby precisely regulating the transcriptional activation state of genes, which is particularly manifested in the sustained enhancement effect on the KRAS signal transduction cascade.^[^ ^34^ ^]^

Quantitative analysis of gene expression changes induced by transcription factor synergistic actions is typically achieved through high‐throughput RNA sequencing (RNA‐seq) or real‐time quantitative PCR (qPCR),^[^ ^35^ ^]^ while precise measurement of chromatin accessibility primarily relies on assay for transposase‐accessible chromatin sequencing (ATAC‐seq) and chromatin immunoprecipitation sequencing (ChIP‐seq) technologies. Specifically, ATAC‐seq can systematically assess the dynamic changes in chromatin open states across the whole genome, while ChIP‐seq can precisely locate the specific binding sites and binding affinities of transcription factors throughout the genome, thereby enabling comprehensive evaluation of their functional contributions in transcriptional co‐regulatory networks.^[^ ^36^ ^]^

### 2.2 Signaling Pathway‐Mediated Synergy–Antagonism

Interactions between intracellular signaling pathways constitute the fundamental molecular basis for synergistic or antagonistic effects. Different signaling pathways can influence each other through activation, inhibition, or feedback regulatory mechanisms: specifically, the activation of one signaling pathway may enhance signal transduction in another pathway, thereby producing synergistic effects.^[^ ^37^ ^]^ Conversely, some pathways may exhibit antagonistic effects by inhibiting key components of other pathways. These complex interactions between signaling networks play crucial roles in regulating fundamental biological processes such as cell growth, differentiation, and survival.^[^ ^38^ ^]^

Abdallah et al. found that the combination of pazopanib and metformin effectively inhibits tumor cell proliferation by suppressing signaling pathways such as phosphorylated Akt/ nuclear factor kappa‐light‐chain‐enhancer of activated B cells/ signal transducer and activator of transcription 3 (p‐Akt/NF‐κB/IL‐6/STAT3) and hypoxia‐inducible factor 1‐alpha/ vascular endothelial growth factor/ (HIF1α/VEGF), thereby reducing the activity levels of Akt and NF‐κB, decreasing Interleukin‐6 (IL‐6) secretion, and inhibiting STAT3 signal transduction, which collectively weakens the pro‐inflammatory microenvironment. Additionally, this drug combination reduces the expression levels of HIF1α and VEGF, inhibits tumor angiogenesis, and limits blood supply to tumor tissues, ultimately leading to tumor cell apoptosis and significantly enhancing the therapeutic effect against lung cancer.^[^ ^39^ ^]^ Zhang et al.’s research demonstrates that enhancer of Zeste homolog 2 (EZH2) and euchromatic histone lysine methyltransferase 2 (EHMT2) act synergistically in non‐small cell lung cancer (NSCLC), with their interaction leading to the silencing of the tumor suppressor gene mothers against decapentaplegic homolog 4 (SMAD4). The silencing of SMAD4 further activates the extracellular signal‐regulated kinase/ Myelocytomatosis viral oncogene homolog (ERK/c‐Myc) signaling cascade, which plays a pivotal role in the molecular mechanism of drug resistance in NSCLC.^[^ ^40^ ^]^

Furthermore, EZH2 mediates histone Histone H3 lysine 27 (H3K27) trimethylation (H3K27me3) and recruits the Polycomb repressive complex Polycomb Repressive Complex 1(PRC1), which inhibits the transcriptional activity of target genes, thereby enhancing DNA damage repair capability, promoting tumor cell proliferation, and inhibiting the apoptotic process, ultimately leading to the development of platinum drug resistance.^[^ ^41^ ^]^

### 2.3 Membrane Transport Protein‐Mediated Synergy–Antagonism

Membrane transport proteins constitute a class of functional proteins localized on biological cell membranes, whose primary function is the transfer of various molecules between the intracellular and extracellular environments. Through specific conformational changes, these proteins precisely facilitate the transport of substrates across the cellular membrane. During molecular transport, membrane transport proteins undergo a series of highly orchestrated conformational changes, including inward‐open, substrate‐bound, and outward‐open critical conformational states. When substrates bind to specific binding sites, these proteins undergo significant conformational changes, leading to the closure of internal sites and the opening of the external side, thereby facilitating the directional release of substrates across the membrane.^[^ ^42^ ^]^

This precise transport process is typically regulated by energy input, predominantly through ATP hydrolysis. Substrate specificity is primarily determined by specific amino acid residues at binding sites, which recognize particular substrates through various non‐covalent interactions, including hydrogen bonds, electrostatic interactions, and hydrophobic interactions, whereas selective pores in the protein structure further ensure the high selectivity of substrate transport.^[^ ^43^ ^]^ In recent years, the development of membrane transport protein inhibitors has yielded significant advances in the treatment of tumors and metabolic diseases, manifesting particularly in the following aspects: First, P‐glycoprotein inhibitors have been extensively studied to overcome multidrug resistance phenomena in cancer; second, GLUT inhibitors significantly reduce the abnormal glucose uptake by tumor cells, thereby interfering with their energy metabolism; Additionally, ABC transporter inhibitors enhance the therapeutic efficacy of anticancer drugs by inhibiting drug efflux and circumventing resistance transport mechanisms.^[^ ^44^, ^45^ ^]^

P‐glycoprotein (P‐gp)‐mediated drug interactions represent the most widely documented and thoroughly studied example of membrane transport proteins influencing drug effects.^[^ ^30^, ^44^, ^46^, ^49^, ^94^ ^]^ P‐glycoprotein is an ATP‐dependent transmembrane transporter that functions by actively effluxing structurally diverse drug molecules from cells, thereby significantly affecting their in vivo distribution, bioavailability, and effective concentrations within target organs.^[^ ^46^ ^]^ When certain drugs act as inhibitors of P‐glycoprotein transport function, they can increase the intracellular accumulation of other co‐administered drugs, thereby producing potential synergistic effects or enhanced toxicity.^[^ ^46^ ^]^ Conversely, when multiple drugs competitively bind to P‐glycoprotein binding sites simultaneously, this competition may lead to decreased intracellular concentrations of certain drugs, reducing their therapeutic efficacy and ultimately resulting in therapeutic antagonism. This transport protein‐mediated drug interaction is of particularly significant clinical relevance in the co‐administration of antitumor chemotherapeutic drugs and antibiotics.^[^ ^47^ ^]^

In studies predicting drug effects related to transport proteins, the MCNN‐DDI deep learning model developed by Asfand‐E‐Yar et al. integrates chemical structure information of aspirin and warfarin simultaneously, along with related metabolic enzyme and transport protein data, while incorporating the specific influence mechanisms of these drugs on coagulation pathways and platelet function, to successfully predict potentially severe adverse interactions between these two medications.^[^ ^48^ ^]^ This type of prediction model provides valuable guidance for clinical practice, not only helping physicians adopt more cautious strategies when prescribing combination drug regimens but also effectively reducing the likelihood of patients experiencing serious adverse reactions through precise risk assessment of drug interaction mechanisms.^[^ ^48^ ^]^

## 3 Applications of Protein Spatial Structures in Drug Synergy–Antagonism Research

The precise elucidation of three‐dimensional protein structures holds significant scientific importance for investigating drug synergistic and antagonistic effects. Through accurate drug binding site specificity recognition analysis and high‐throughput molecular docking technologies, researchers can systematically and thoroughly understand the molecular‐level interaction mechanisms between drugs and targets, thereby facilitating further investigations into the mechanisms of drug synergistic and antagonistic effects.^[^ ^12^ ^]^ Multi‐target drug strategies enhance the therapeutic efficacy of drug combinations and reduce the risk of resistance by simultaneously and specifically acting on functional targets in multiple key signaling pathways. Computer‐aided virtual screening and de novo drug design methods based on high‐resolution three‐dimensional protein structures effectively utilize protein–ligand interaction interface characteristics,^[^ ^23^ ^]^ significantly facilitating the discovery and rational design of novel targeted drugs, substantially accelerating drug development processes, and reducing development costs.

### 3.1 Structural Basis of Protein–Drug Interactions

#### 3.1.1 Identification and Characterization of Drug Binding Sites

Biomacromolecular surfaces typically contain sites capable of specific ligand binding, with protein–ligand interactions being primarily mediated by amino acid residues at specific positions,^[^ ^49^ ^]^ which are usually located in concave regions on the protein surface, termed ligand binding sites (LBS).^[^ ^50^ ^]^ Binding sites are primarily classified into two types: i) active sites, where substrates, transition states, products, and various competitive inhibitors can bind, thereby facilitating the catalytic function of the target protein or inhibiting its activity;^[^ ^50^, ^51^ ^]^ ii) allosteric sites, or regulatory sites, where ligand binding may enhance or inhibit protein function. Ligand binding to allosteric sites of multimeric enzymes often induces positive cooperativity, in which the binding of one substrate promotes favorable conformational changes in the protein, thereby increasing the enzyme's affinity for subsequent substrates.^[^ ^52^ ^]^ Upon binding to specific protein sites, ligands can induce conformational changes in proteins, leading to the modulation of protein function and ultimately affecting the biological processes in which the targets participate.^[^ ^51^, ^53^ ^]^ In recent research, methods for predicting ligand binding sites have been primarily divided into two categories: sequence‐based methods and structure‐based methods.^[^ ^54^ ^]^ Sequence‐based methods primarily identify sites with specific structural or functional characteristics by analyzing the conservation of key amino acid residues across protein sequences. Common tools in this category include algorithms such as Firestar (CASP9 – group FN315),^[^ ^55^, ^56^ ^]^ WSsas,^[^ ^57^ ^]^ and ConFunc (CASP8 – FN437).^[^ ^58^ ^]^ Structure‐based methods can be further subdivided into: geometric feature methods^[^ ^59^, ^60^ ^]^ (such as FINDSITE and Surflex‐PSIM), energy calculation methods^[^ ^61^ ^]^ (such as SITEHOUND), and comprehensive analysis methods that integrate multiple features. These comprehensive methods primarily employ technologies such as homology modeling (e.g., FunFOLD – CASP9 FN425^[^ ^62^ ^]^), surface accessibility analysis (e.g., LIGSITE CSC^[^ ^63^ ^]^), and physicochemical property assessment (e.g., SCREEN^[^ ^64^ ^]^). These methods provide diverse computational approaches for high‐precision prediction of ligand binding sites in drug development by integrating sequence conservation, protein geometry, and relevant physicochemical properties. To systematically compare and analyze the aforementioned tools and methods, this study comprehensively summarizes them across multiple dimensions, including algorithm implementation characteristics, computational performance, and application scope (**Table** **1**).

**Table 1:** Comparative analysis of ligand binding site prediction tools: features and performance metrics.

Method category	Method name	Implementation characteristics	Computational performance	Scope of application
Sequence‐based Methods	Firestar	FireDB database to provide protein–ligand binding information; utilizes PSI‐BLAST and HHsearch for sequence alignment and functional residue matching; incorporates SQUARE evaluation to ensure prediction reliability; offers a fully automated prediction pipeline supporting high‐throughput analysis and Web service integration.	Demonstrates superior performance in CASP7, CASP8, and CASP9 assessments with high MCC scores; integration with HHsearch extends sequence alignment capabilities, improving prediction coverage by 34%.	Applications include functional residue prediction; drug design and computational screening; protein function annotation; structural biology research; systems biology & genomic research.^[^ ^55^ ^]^
	WSsas	Based on structural homology; predicts protein functional residues; integrates PDBsum and CSA data to ensure annotations are based on resolved structures; supports batch analysis and Web service API (SOAP protocol) suitable for high‐throughput functional annotation.	Applicable for large‐scale sequence analysis; features flexible threshold adjustment; utilizes 3D structural information for precise function transfer, outperforming sequence‐only methods.	Applications include protein functional residue prediction; drug discovery and target screening; protein function annotation; structural biology; computational biology.^[^ ^57^ ^]^
	FRcons	Predicts protein functional residues based on probability density estimation; integrates sequence conservation, amino acid distribution, secondary structure, and solvent accessibility; employs conditional probability estimation to improve functional site prediction accuracy; utilizes neighboring residue window methods to optimize signal‐to‐noise ratio, enhancing prediction reliability; validated across multiple datasets.	Achieves 50% precision for ligand binding sites in CSA‐ligand dataset tests; outperforms traditional conservation methods, including entropy, relative entropy, JSD variations, and Rate4Site.	Applications include protein functional residue prediction; drug target screening; evolutionary analysis; systems biology; structural biology.^[^ ^159^ ^]^
	ConFunc	Performs protein function prediction based on GO annotations; uses PSI‐BLAST to identify homologous sequences; conducts sub‐alignments according to GO terms; analyzes conserved residues combined with PSSM to calculate GO‐related conserved sites; an automated workflow applicable to large‐scale genomic functional annotation; employs Vingron‐type sequence weighting to reduce evolutionary distance bias.	Achieves recall rates far exceeding traditional sequence alignment methods under high precision conditions, performing well even in the absence of closely related homologous sequences.	Applications include protein function prediction; GO term assignment; evolutionary conservation analysis; large‐scale bioinformatics research.^[^ ^58^ ^]^
	ConSurf	Predicts functionally critical sites based on evolutionary conservation analysis; calculates evolutionary rates using Bayesian inference and maximum likelihood (ML) methods; integrates protein sequences with 3D structures to visualize conservation scores; identifies homologous sequences via BLAST/PSI‐BLAST with CD‐HIT filtering to remove redundant sequences.	Employs Rate4Site to calculate evolutionary rates combining Bayesian and ML estimates; incorporates multiple substitution models; features high‐throughput data processing with automatic removal of low‐quality alignments; utilizes a 9‐grade conservation scoring system (grade 1: highly variable, grade 9: highly conserved).	Applications include protein functional site prediction; protein‐nucleic acid interaction analysis; structural biology; evolutionary analysis; systems biology.^[^ ^160^ ^]^
	FPSDP	Predicts protein functional sites based on evolutionary analysis & 3D structural information; features automated database collection of functional residue patterns from homologous protein families; calculates amino acid conservation using environment‐dependent substitution tables; identifies highly conserved functional residues through evolutionary trace analysis; assesses functional residue availability for interactions through solvent accessibility calculations; extracts patterns from HOMSTRAD structural alignment database to improve accuracy.	Performs better in enzyme active site recognition compared to protein–protein binding pattern identification; achieves more precise pattern recognition for α‐helical proteins, with slightly more challenging identification for β‐sheet proteins.	Applications include protein function prediction; protein family classification; drug target screening; protein–protein interaction studies; biomedical research.^[^ ^161^ ^]^
	INTREPID	Predicts protein functional residues based on evolutionary tree traversal methods, analyzing sequence conservation patterns; employs information theory scoring systems to evaluate amino acid functional importance across different evolutionary subtrees; determines conserved sites through multiple sequence alignment + phylogenetic tree analysis, avoiding oversight of locally important residues due to global variations; features automated data processing; supports visualization with display of predicted important residue sites on homologous 3D structures.	Improves recall rate and precision of catalytic site prediction; applicable to highly divergent protein families, enhancing diversity adaptation capabilities; offers high computational efficiency (complete analysis <15 minutes, scoring calculation <5 minutes).	Applications include functional residue prediction; protein evolution analysis; protein function annotation; drug target identification; computational biology research.^[^ ^162^ ^]^
	SS‐TEA	Identifies G protein‐coupled receptor (GPCR) specific ligand binding residues based on entropy analysis methods; utilizes large‐scale multiple sequence alignments (MSA) to identify evolutionary conservation and receptor‐specific binding sites; employs subfamily‐level two‐entropy analysis (TEA) to improve ligand binding site precision; integrates 13324 non‐olfactory class A GPCR sequences, providing the most comprehensive cross‐species MSA; offers visualization analysis & data access through the GPCRDB online database.	Capable of identifying unique binding sites across different receptor subfamilies; features high computational efficiency applicable to large‐scale sequence analysis; incorporates evolutionary distance analysis to ensure accuracy of receptor‐specific binding sites.	Applications include GPCR receptor ligand binding site prediction; drug design and computational screening; protein function annotation; structural biology; systems biology.^[^ ^163^ ^]^
Structure‐based Methods	FINDSITE (Geometric method)	Predicts ligand binding sites and performs functional annotation based on Threading methods; enhances prediction reliability through PROSPECTOR_3 for ligand binding template identification; employs TM‐align structural alignment to superimpose multiple templates onto target proteins, identifying consensus binding sites; tolerates structural errors, capable of processing protein models with below 35% sequence homology; incorporates ligand chemical information, extracting binding site chemical properties from template structures for virtual screening.	Achieves a 67.3% success rate for low‐homology protein model predictions; tolerates structural errors of 8–10 Å, with most predicted binding sites having root mean square deviation (RMSD) <2 Å.	Applications include protein–ligand binding site prediction; drug target screening; functional annotation; evolutionary analysis research on functional convergence and protein evolution.^[^ ^59^ ^]^
	Surflex‐PSIM (Geometric method)	Protein binding site comparison based on 3D morphological similarity; local pocket alignment combining protein surface morphology and polarity characteristics; automated alignment optimization to reduce solvent accessibility errors; Surflex‐PSIM module supports protein pocket construction and ligand activity prediction; analysis at the all‐atom level.	Demonstrates superior performance on protein kinase datasets; high computational efficiency with capability to automatically generate global protein similarity trees.	Protein–ligand binding site analysis; drug discovery and computational screening; protein function annotation; evolutionary biology research; computational biology.^[^ ^60^ ^]^
	SITEHOUND (Energy‐based method)	Identifies protein–ligand binding sites based on energy calculations; probe molecules scan protein surfaces to predict binding regions; supports multiple probe types; hierarchical clustering analysis identifies and ranks high‐energy interaction sites; binding site visualization (supporting Jmol3D interaction and PyMOL analysis).	High accuracy in binding site prediction; short computation time; supports automated PDB processing; ranks binding sites based on TIE (Total Interaction Energy) to screen the most probable binding regions.	Protein–ligand binding site prediction; drug design and computational screening; protein function annotation; structural biology; systems biology.^[^ ^61^ ^]^
	FunFOLD (Homology modeling)	Predicts protein–ligand binding sites based on 3D structure superposition alignment; automated ligand clustering and residue selection to avoid manual parameter adjustment; TM‐align for template structure screening to ensure prediction quality; ModFOLDclust2 selects optimal 3D structural models to improve prediction accuracy.	Employs Binding‐site Distance Test (BDT) scoring, providing more rigorous binding site prediction assessment; performs well in metal binding site prediction.	Protein–ligand binding site prediction; drug target identification; protein function prediction; computational biology and biomedical research; high‐throughput automated analysis.^[^ ^62^ ^]^
	3DLigandSite (Homology modeling)	Predicts ligand binding sites based on structural similarity; MAMMOTH structural scanning identifies similar proteins; supports protein sequence or structure input; Phyre server predicts protein structure; single‐linkage clustering method processes ligands; Jmol visualization for result display.	Prediction accuracy varies with ligand distance threshold (0.2Å‐2.0Å), with optimal prediction performance at 0.8Å threshold reaching MCC 0.68, 70% accuracy, and 70% coverage.	Ligand binding site prediction; drug target research; function annotation; structural comparative analysis.^[^ ^164^ ^]^
	I‐TASSER_FUNCTION (Homology modeling)	Predicts protein structure based on Threading, Monte Carlo simulation, and energy optimization; LOMETS identifies templates and TASSER fragment assembly generates 3D structures; TM‐align performs structural alignment to predict protein function.	Performs best in CASP assessments; can process proteins of 10–1500 amino acids; RMSD error range of 2Å, with high prediction accuracy as measured by TM‐score.	Protein 3D structure prediction; function annotation; protein family classification; drug screening; systems biology.^[^ ^165^ ^]^
	LIGSITE CSC (Surface accessibility)	Predicts ligand binding sites based on Connolly surface and residue conservation; improves LIGSITE algorithm by employing SSS (Surface‐Solvent‐Surface events replacing PSP Protein‐Solvent‐Protein) events; multi‐directional scanning (X, Y, Z axes & 4 cubic diagonals) enhances identification precision; automated pocket reranking, integrating evolutionary conservation to reprioritize binding sites.	Conservation score reranking improves prediction precision; can process large‐scale protein–ligand binding datasets; compared to PASS and SURFNET, reduces false positive rates and enhances accuracy.	Protein–ligand binding site prediction; drug screening; function annotation; computational biology; evolutionary biology.^[^ ^63^ ^]^
	SCREEN (Physicochemical properties)	Characterizes and maps protein–ligand binding sites based on Principal Component Analysis (PCA); does not rely on geometric alignment, analyzing binding sites through physicochemical properties; automated protein cavity detection identifies solvent‐accessible regions on protein surfaces and calculates properties; utilizes 408 surface descriptors to compute size, shape, polarity, electrostatic field, and other properties; PCA generates clustering trees for protein structure selection, docking studies, drug design, and more.	High success rate in binding site prediction; can differentiate proteins with similar structures but different functions, improving target selection precision; fast computation speed.	Protein–ligand binding site prediction; drug discovery and structural biology; protein function annotation; systems biology and computational biology; biomedical research.^[^ ^64^ ^]^

Abbreviations: FireDB: functional information resource for enzymes database, PSI‐BLAST: position‐specific iterated basic local alignment search tool, HHsearch: hidden Markov Models‐ Hidden Markov Models Search, SQUARE: Sequence Quality Assessment by Residue Evaluation, MCC: Matthews Correlation Coefficient, CASP: Critical Assessment of protein Structure Prediction, PDBsum: Protein Data Bank Summary, CSA: Catalytic Site Atlas, JSD: Jensen‐Shannon Divergence, GO: Gene Ontology, CD‐HIT: Cluster Database at High Identity with Tolerance, HOMSTRAD: HOMologous STRucture Alignment Database, Surflex‐PSIM: Surflex‐based Pocket Similarity Identification Method, MAMMOTH: Matching Molecular Models Obtained from Theory, LOMETS: Local Meta‐Threading Server, PASS: Putative Active Site with Spheres, SURFNET: Surface Network.

Further analysis of Table 1 indicates that sequence‐based methods offer advantages such as minimal resource requirements and their applicability to proteins lacking experimentally determined structures, thereby facilitating the identification of highly conserved potential binding sites. However, these methods exhibit lower prediction accuracy when applied to proteins exhibiting significant structural diversity and encounter challenges in identifying non‐conserved regions, such as allosteric sites. Structure‐based methods demonstrate superior performance in the identification of spatial pockets and in energy optimization, wherein geometry‐based tools such as FINDSITE and Surflex‐PSIM are particularly suitable for large‐scale screening; however, they face limitations when handling flexible conformations or low‐resolution structures. Although energy calculation methods and integrated feature approaches offer enhanced predictive power, they are computationally expensive and dependent on the availability of complete and high‐quality protein structures. In practical applications, the accuracy of these prediction tools requires validation by experimental methods, such as mutational analysis and affinity assays. In summary, different methods exhibit distinct advantages and limitations in terms of their scope of application, precision, and interpretability, and a universally optimal solution has yet to emerge. Therefore, the rational selection of tools tailored to specific research requirements, or the integration of multiple predictive methods for complementary optimization, is more likely to yield high‐confidence predictions of ligand‐binding sites and to provide a solid foundation for structure‐based drug design.

#### 3.1.2 Molecular Docking Techniques in Drug–Target Interaction Prediction

Molecular docking is a well‐established computational structural method widely applied in drug discovery that identifies novel therapeutic compounds by predicting the interaction modes between ligands and targets. Molecular docking techniques simulate the binding process between small molecules (ligands) and macromolecular targets (receptors), aiming to predict their binding affinities and optimal binding conformations, thereby identifying plausible low‐energy interaction modes within the active site. It is important to note that the precision of these predictions can be influenced by the simplified models and the inherent inaccuracies in the scoring functions employed. With the ongoing advancement of computational technologies and algorithms, numerous efficient and diverse molecular docking software platforms have emerged. Different docking software platforms exhibit distinct characteristics in computational accuracy, algorithmic efficiency, and application scope, and have been widely implemented in drug design, virtual screening, target identification, and drug optimization. Herein, we systematically summarize and compare these methods across multiple dimensions, including model types, application scopes, computational complexity, and their respective advantages and disadvantages. (**Table** **2**)

**Table 2:** Comparison of molecular docking tools: model types, application scope, computational complexity, and advantages and disadvantages.

Method name	Applicable scope	Calculation complexity	Advantages	Limitations
**AutoDock**	Protein–small molecule ligand docking; protein–protein docking; nucleic acid–ligand docking; metal cofactor binding prediction; covalent ligand docking.	Medium	Free and open‐source, compatible with multiple operating systems; supports partially flexible receptor docking; AutoGrid pre‐calculates grid energies to enhance computational efficiency and reduce redundant calculations; supports large‐scale virtual screening; integrates AutoDockTools (ADT) providing a visual interface for convenient parameter setting and result analysis.	Limited capability in receptor flexibility modeling, unable to handle large‐scale protein conformational changes; relatively long computation time; accuracy depends on energy function parameters, requiring user optimization to improve precision; lacks explicit solvent models, with calculations defaulting to vacuum environments, potentially affecting accuracy under physiological conditions.^[^ ^65^ ^]^
**AutoDock Vina**	Protein–small molecule ligand docking; nucleic acid–small molecule docking; metal cofactor binding prediction; macromolecular substrate docking.	Low	Faster computation than AutoDock4, suitable for high‐throughput screening; built‐in multi‐threading support, enabling simultaneous calculation of multiple docking tasks; more accurate binding conformation predictions with higher success rates when combined with experimental data; greater applicability for macromolecular substrates; faster convergence of computational results, suitable for resource‐constrained computing environments.	Binding energy scoring is less precise than AutoDock4, with significant prediction biases in certain systems; limited support for receptor flexibility, unable to handle large‐scale protein conformational changes; cannot accurately estimate binding free energy, with errors in some binding energy calculations; not suitable for high‐precision protein–protein docking; performs poorly with specific receptors (such as Glutaminyl Cyclase and β‐amyloid peptides).^[^ ^66^ ^]^
**DEL‐Dock**	DNA‐encoded library (DEL) molecular screening; integration of molecular docking with DEL data modeling; prediction of small molecule‐protein binding affinity; applicable for large‐scale virtual screening.	Relatively High	Combines docking data to improve affinity prediction accuracy; automatically learns docking site information; trained on DEL data, not dependent on expensive crystal structure data; demonstrates superior ranking performance compared to traditional docking methods.	Only applicable to proteins with crystal structures; docking results depend on the accuracy of docking software; unable to directly predict binding modes for unknown targets; does not consider protein flexibility, potentially affecting the precision of certain ligand docking predictions.^[^ ^67^ ^]^
**CB‐Dock2**	Blind docking; binding pocket detection; receptor‐ligand interaction prediction; computational drug design.	Low	Combines two docking approaches (structure‐template binding, improving precision); supports interactive 3D result analysis; optimized user interface, lowering usage barriers; supports ligand drawing and structure uploading.	Unable to distinguish between biological units and asymmetric units; does not support protein flexibility modeling and missing residue optimization; docking engine still requires improvement, with potential for future enhancement.^[^ ^68^ ^]^
**Glide(Schrodinger)**	Drug discovery, virtual screening, receptor‐ligand interaction studies, biomedical research, molecular simulation.	Medium to relatively high	High precision, outperforming many competitive methods in docking and scoring; strong flexibility, applicable to various types of receptors (proteins) and ligands; suitable for large‐scale virtual screening; physicochemical constraints; improved docking accuracy combined with the optimized scoring function GlideScore.	High computational cost, especially in Extra Precision mode, requiring substantial computational resources; limited protein flexibility handling; modeling of hydrophobic binding sites still needs improvement; requires parameter optimization.^[^ ^166^ ^]^
**GlycanDock**	Protein‐glycan ligand docking optimization; glycoprotein structure prediction; binding pocket prediction and affinity optimization; computational glycobiology research.	Relatively high	Allows glycan ligand flexibility optimization; considers glycosidic bond torsional degrees of freedom, improving docking precision; integrates with the RosettaCarbohydrate framework, supporting complex glycan chains; high‐resolution scoring function, enhancing binding mode prediction accuracy; suitable for high‐throughput computation.	Unable to simulate protein backbone flexibility; docking results limited by input structure quality; water‐mediated hydrogen bonds difficult to model precisely; some glycan‐protein interactions may not be fully captured.^[^ ^70^ ^]^
**HADDOCK**	Protein‐carbohydrate complex modeling; High Ambiguity Driven Docking; Experiment‐based molecular docking; Computational drug discovery and glycoconjugate analysis.	High	Integration of experimental data for optimized docking; Support for flexible carbohydrate ligand modeling; Modular design allowing user‐customized optimization workflows; Applicable for protein–protein, protein–small molecule, and protein‐carbohydrate interaction predictions; Incorporation of advanced scoring functions to enhance docking accuracy.	Decreased docking success rates with highly flexible carbohydrates; Requires prior knowledge of binding pocket information, otherwise predictions become challenging; Computationally intensive, suitable for high‐performance computing environments; Potentially unstable ranking performance for certain long‐chain carbohydrates.^[^ ^69^ ^]^
**Gold**	Molecular docking; Computational chemistry and drug discovery; Protein–ligand interaction analysis; Molecular design.	Medium	Supports ligand flexibility optimization; Hydrophobic optimization of binding pockets; Multiple scoring functions; Applicable to various protein targets; Stable results, compatible with visualization tools.	Computationally intensive, dependent on high‐performance computing; Average performance for polar binding pockets; Lacks support for metal coordination modeling; Requires manual parameter depth adjustment.^[^ ^167^ ^]^

A more rigorous, multi‐dimensional comparison of the tools presented in Table 2 was undertaken to comprehensively assess their applicability and reliability. Significant disparities are evident among different docking methods regarding computational complexity, predictive accuracy, capacity for handling molecular flexibility, and dependence on experimental data. The AutoDock suite of tools, a classic open‐source docking platform, offers considerable flexibility and reproducibility; however, its accuracy is limited when modeling large‐scale conformational changes in receptors.^[^ ^65^ ^]^ Although AutoDock Vina demonstrates advantages in speed and high‐throughput screening capabilities, its energy scoring function exhibits certain biases, thereby impacting the accurate assessment of binding free energies.^[^ ^66^ ^]^ Emerging tools such as DEL‐Dock and CB‐Dock2 have enhanced model precision by integrating high‐throughput library information with binding pocket predictions, making them suitable for large‐scale screening tasks; however, their applicability to unknown targets and proteins lacking crystal structures remains restricted.^[^ ^67^, ^68^ ^]^ For specific target classes, such as glycan‐binding proteins, highly specialized platforms like GlycanDock and HADDOCK offer optimized support for handling the flexibility of glycan ligands and their bond torsion angles; nevertheless, these platforms require substantial high‐performance computing resources, and the accuracy of their predictions is sensitive to the quality of the input structures.^[^ ^69^, ^70^ ^]^ Overall, a disparity persists between the theoretical predictive performance of molecular docking tools and their practical effectiveness in actual biological systems; consequently, experimental validation of binding affinity remains paramount for evaluating their real‐world performance. Future research efforts should prioritize the development of docking engines capable of simultaneously balancing computational efficiency and the modeling of conformational flexibility, and progressively integrate synergistic optimization strategies that combine artificial intelligence with molecular dynamics to improve the generalizability and biological interpretability of predictions.

### 3.2 Structural Mechanism Studies of Multi‐Target Drug Actions

#### 3.2.1 Structural Basis of Multi‐Target Drug Synergistic Effects

Multi‐target drugs refer to a class of pharmaceuticals that enhance overall therapeutic efficacy by acting on multiple distinct targets or several key nodes within the same signaling pathway. At the molecular level, the targets of these multi‐target drugs may be located within the same protein structure or distributed across different protein entities. These drugs achieve enhanced therapeutic effects by interacting with multiple binding sites and synergistically regulating diverse signaling pathways or biological processes.^[^ ^9^ ^]^ Currently, prominent multi‐target drugs in clinical applications are primarily concentrated in therapeutic areas such as anticancer therapy, cardiovascular disease treatment, and neurological disorders,^[^ ^71^ ^]^ with notable examples including the anticancer drug sorafenib. Sorafenib, as a multi‐target inhibitor, exerts anticancer effects through several interconnected synergistic mechanisms. First, sorafenib inhibits tumor cell proliferation by suppressing c‐Raf kinase and its downstream signaling pathways, thereby blocking MEK and ERK phosphorylation and consequently reducing ERK activation levels. Second, this drug effectively inhibits tumor angiogenesis by suppressing the autophosphorylation activities of tyrosine kinase receptors, including VEGFR‐2, VEGFR‐3, and PDGFR‐β.^[^ ^72^ ^]^ Beyond the aforementioned mechanisms, sorafenib also inhibits the phosphorylation of translation initiation factor eIF4E, thereby downregulating the expression levels of anti‐apoptotic protein Mcl‐1 and subsequently promoting tumor cell apoptosis.^[^ ^73^ ^]^ HDAC/EZH2 dual‐target inhibitors designed based on the “dual targets in a single drug” strategy have significantly improved therapeutic outcomes for hematological malignancies, particularly acute myeloid leukemia and diffuse large B‐cell lymphoma.^[^ ^74^ ^]^ In the field of neurological disorders, Chen et al. demonstrated that IHCH‐7179, developed through a flexible molecular scaffold design strategy, simultaneously targets two different receptors: functioning as a 5‐HT2AR antagonist to alleviate psychiatric symptoms and as a 5‐HT1AR agonist to improve cognitive function, thereby exhibiting significant potential in treating dementia‐related psychosis.^[^ ^75^ ^]^ Psilocybin (LSD), as a classic multi‐target drug, acts on multiple 5‐HT serotonin receptors, particularly exerting strong agonistic effects on the 5‐HT2A receptor, which induces psychiatric symptoms such as hallucinations, while its synergistic effects on other receptors confer complex and diverse pharmacological properties.^[^ ^76^ ^]^ Additionally, widely used atypical antipsychotics (such as clozapine and aripiprazole) achieve antipsychotic effects synergistically through selective interactions with various G protein‐coupled receptors (GPCRs). Compared to traditional single‐target drugs, these multi‐target drugs significantly reduce extrapyramidal side effects and offer superior efficacy in treating psychiatric disorders such as schizophrenia.^[^ ^77^ ^]^

#### 3.2.2 Structural Mechanisms of Multi‐Target Drug Antagonistic Effects

During combined administration, drugs may reduce overall therapeutic efficacy by acting on different targets or interfering with the mechanistic activity of the same target. These drug interaction mechanisms can be categorized into three primary types: i) competitive inhibition, where multiple drugs mutually inhibit each other by competing for the same receptor or binding site, or inhibit their respective metabolic processes by competing for the same metabolic enzyme, resulting in significantly diminished drug efficacy. Sulfonamide antibacterial drugs (SAs) represent classic examples of this competitive inhibition mechanism. Due to the high structural similarity between SAs and para‐aminobenzoic acid (PABA), the natural substrate of dihydrofolate synthase, when the in vivo concentration of SAs substantially exceeds that of PABA, they preferentially occupy the substrate binding site of the enzyme, thereby blocking enzymatic utilization of PABA for dihydrofolate synthesis.^[^ ^78^ ^]^ Another example is the anti‐influenza drug oseltamivir, which competitively inhibits influenza virus neuraminidase activity by occupying the enzyme's active site, thereby effectively blocking the binding and conversion of natural substrates.^[^ ^79^ ^]^ ii) Negative feedback regulatory mechanisms of signaling pathways,^[^ ^80^ ^]^ which refer to compensatory pathway activation induced following drug intervention; For example, B56γ3 protein simultaneously positively regulates AKT phosphorylation while negatively regulating p70S6K phosphorylation. By attenuating the negative feedback regulation mediated by downstream p70S6K under growth factor stimulation, this protein upregulates AKT phosphorylation levels, thereby enhancing AKT activity, promoting epithelial‐mesenchymal transition (EMT), and significantly reducing the sensitivity of colorectal cancer cells to therapeutic drugs.^[^ ^81^ ^]^ iii) Drugs interfering with each other's biotransformation processes, resulting in significantly weakened efficacy or adverse reactions. A representative case is the proton pump inhibitor omeprazole, which is primarily metabolized by the CYP2C19 subtype of the cytochrome P450 enzyme system. When co‐administered with other drugs that utilize the same enzyme system for metabolism, such as diazepam and phenytoin, omeprazole competitively inhibits their metabolism and significantly prolongs their metabolic clearance time, thereby affecting their clinical efficacy.^[^ ^82^ ^]^

### 3.3 Applications of Structure‐Based Drug Design in Synergistic–Antagonistic Research

#### 3.3.1 Structure‐Based Virtual Screening

Structure‐Based Virtual Screening (SBVS) is a computational technique for compound screening that has been extensively applied in drug discovery, demonstrating unique advantages, particularly in synergistic‐antagonistic effect research.^[^ ^83^ ^]^ SBVS aims to systematically search and rank the accessible chemical space of potential ligands based on three‐dimensional structural models of target macromolecules (typically proteins or RNA structures), thereby identifying potential drug molecules that may interact with multiple targets, which plays a crucial role, especially in the study of complex synergistic and antagonistic effects.^[^ ^84^ ^]^ In recent years, the application of SBVS in drug discovery has grown exponentially, demonstrating exceptional performance, particularly in the study of complex regulatory networks involving multi‐target synergistic or antagonistic effects. SBVS can efficiently screen binding conformations between candidate molecules from large‐scale compound libraries and targets, thereby identifying potential active molecules (hits) through systematic evaluation of their binding affinities. Molecular Docking is one of the most commonly used techniques in SBVS, which is employed to predict ligand binding poses, characterize protein–ligand interaction strengths, and identify key binding sites, followed by the application of scoring functions to quantitatively evaluate binding affinities and determine ligand conformational priorities.^[^ ^85^ ^]^ However, limitations in the accuracy of existing scoring functions lead to imprecise ranking of binding conformations and significant deviations in binding free energy predictions, consequently hindering SBVS's ability to effectively distinguish between active and inactive compounds, thus impacting screening efficiency and accuracy.^[^ ^86^ ^]^ Wang et al. successfully screened and identified a phthalimide compound using SBVS technology, which was subsequently confirmed as a selective human interleukin‐6 (hIL‐6) antagonist.^[^ ^87^ ^]^ Additionally, SBVS, as a powerful computational tool in drug design, has successfully helped researchers identify potential antagonistic regulatory mechanisms between thyroid‐stimulating hormone receptor (TSHR), transforming growth factor‐β1 (TGF‐β1), and hyaluronic acid, which have critical significance in thyroid eye disease (TED)‐related tissue remodeling processes.^[^ ^88^ ^]^

#### 3.3.2 De Novo Drug Design

De novo drug design encompasses strategies aimed at designing novel molecules with specific pharmacological properties from scratch. Unlike traditional virtual screening, de novo drug design explores a broader chemical space more efficiently, thereby accelerating the discovery of multi‐target candidate molecules. This approach is particularly suitable for identifying drug molecules optimized for synergistic or antagonistic effects.^[^ ^89^ ^]^ De novo drug design methods are primarily categorized into two approaches: structure‐based design and ligand‐based design. Structure‐based de novo drug design (SBDD) is a strategic approach that utilizes three‐dimensional structural information of target molecules to design novel pharmaceutical compounds from scratch. The core principle of this approach involves designing molecules capable of highly specific interactions with targets (typically proteins or RNA) through a comprehensive understanding of their structures, thereby achieving targeted disease treatment.^[^ ^89^ ^]^ Research conducted by Sun et al. demonstrated that the combination of 5–10 µm Olaparib (a PARP1 inhibitor) with 5 µm Crizotinib (a c‐Met inhibitor) exhibited significant antiproliferative activity in the MDA‐MB‐231 cell line (characterized by c‐Met overexpression and normal BRCA), achieving synergistic effects through this combination therapy. Based on these findings, the research team applied the SBDD methodology to design dual‐target PARP1/c‐Met inhibitors.^[^ ^90^ ^]^ However, the SBDD method presents several limitations, including excessive dependence on precise definitions of active sites, oversimplified interaction models, and inadequate accuracy of scoring functions.^[^ ^91^ ^]^ Furthermore, the high computational complexity of these methods further constrains the precision and efficiency of drug design.^[^ ^92^ ^]^ Ligand‐based de novo drug design serves as an important alternative strategy when biological targets lack three‐dimensional structural information, proving particularly valuable for designing candidate molecules for targets with elusive crystal structures.^[^ ^93^ ^]^ This approach offers advantages in terms of wide applicability and abundant available data sources;^[^ ^94^ ^]^ nevertheless, it is constrained by heavy dependence on pharmacophore model quality.^[^ ^95^ ^]^ Construction of such models necessitates assumptions about common binding patterns, while quality assessment presents substantial complexity and methodological challenges.^[^ ^96^ ^]^ Compound libraries generated through this approach can undergo comprehensive evaluation using scoring functions that assess key properties, including bioactivity, synthetic feasibility, metabolic stability, and pharmacokinetics.^[^ ^97^ ^]^ In response to the COVID‐19 pandemic, Ton et al. developed a deep learning platform called “Deep Docking,” which rapidly predicts docking scores and efficiently performs structure‐based virtual screening of billions of molecules.^[^ ^98^ ^]^ Employing this methodology, they systematically screened over one billion compounds from the ZINC15 database and successfully identified 1000 potential ligands targeting the SARS‐CoV‐2 main protease; these candidate inhibitors not only demonstrate significant chemical diversity but also generally possess higher docking scores compared to known inhibitors.

## 4 Applications of Artificial Intelligence in Drug Synergy–Antagonism Prediction

**Figure 2:**
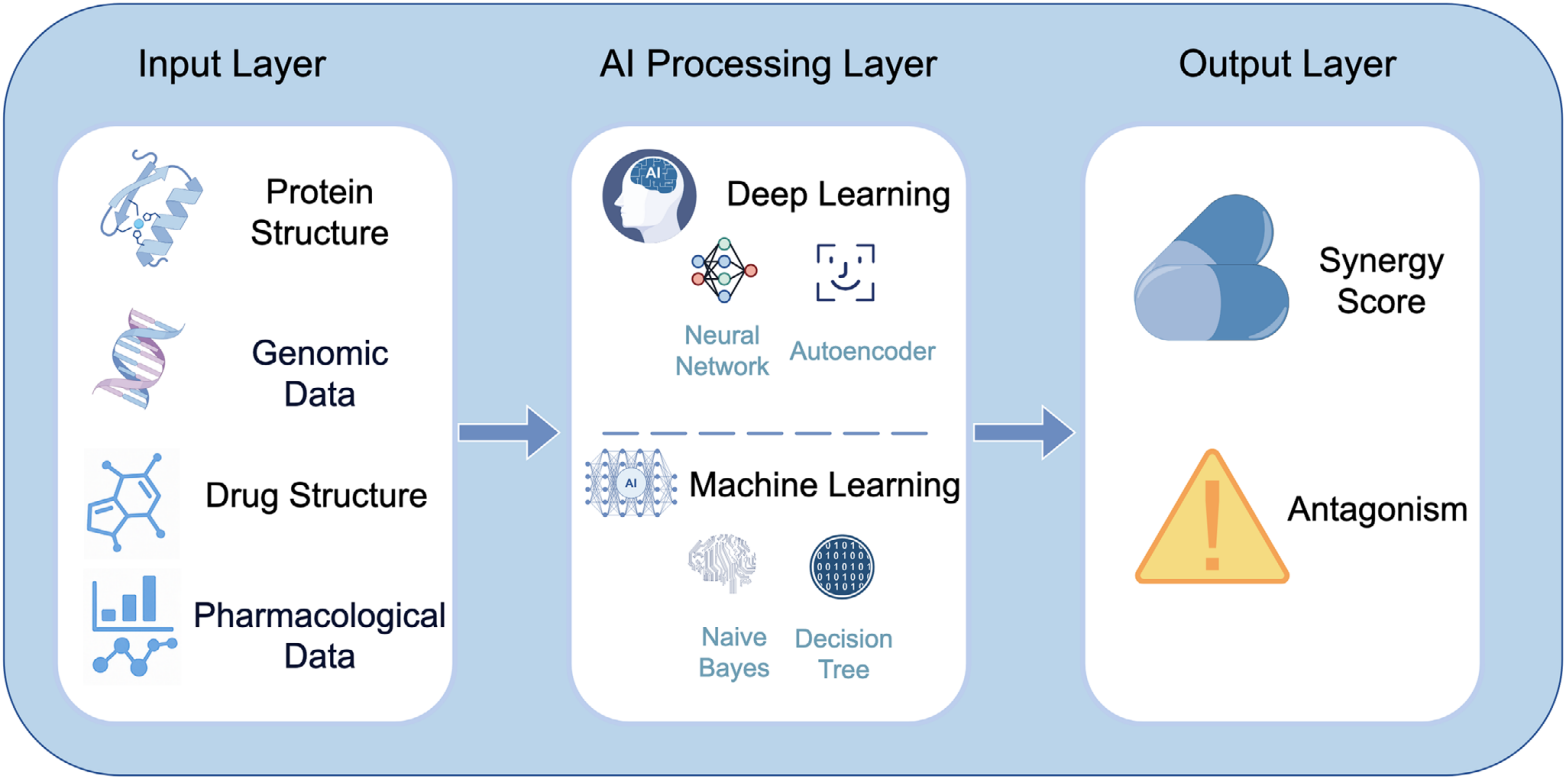
Conceptual workflow for artificial intelligence (AI)‐powered drug synergy–antagonism prediction integrating protein spatial structure data. The schematic diagram depicts a multi‐level workflow that integrates diverse biomedical data sources with AI algorithms to predict drug synergy and toxicity risks. The input layer comprises protein structures, genomic data, drug chemical structures, and established pharmacological properties. Subsequently, these datasets undergo analysis in the AI processing layer, which employs a combination of deep learning techniques (e.g., neural networks, autoencoders) and traditional machine learning algorithms (e.g., Naive Bayes, Decision Trees). The predictive outcomes generated by the output layer, including synergy scores and toxicity risk assessments, can provide a theoretical basis for optimizing drug combinations and guiding clinical decision‐making.

To provide a more systematic and intuitive illustration of the overall technological pathway through which artificial intelligence predicts drug synergy and antagonism, **Figure** **2** presents an AI analytical framework based on biomedical data. This framework encompasses a multimodal input layer, which includes data ranging from three‐dimensional protein structures, drug molecular characteristics, and genomic information, to pharmacokinetic/pharmacodynamic (PK/PD) parameters. This input layer, through joint modeling and feature learning using deep learning and machine learning algorithms, ultimately enables the prediction of synergistic effect scores for drug combinations and the assessment of potential toxicity risks.

### 4.1 Applications of Machine Learning (ML) and Deep Learning (DL) in Synergy–Antagonism Prediction

Machine learning (ML) and Deep learning (DL), as critical branches of computational methodology, have become essential tools in drug synergy–antagonism prediction, effectively optimizing drug mechanisms through the systematic analysis of large biological datasets. Machine learning, through complex data modeling algorithms, effectively identifies interaction patterns between molecules and has been widely applied in various aspects of drug discovery, screening, and target identification. Particularly in the field of drug synergy–antagonism prediction, ML can accurately predict drug interactions and combined effects based on existing drug datasets.^[^ ^17^ ^]^ Deep learning, as an advanced branch of machine learning, demonstrates significant advantages in processing complex high‐dimensional data, efficiently extracting and learning intricate interrelationships between drugs and targets from structurally diverse raw data. Through sophisticated algorithms such as convolutional neural networks (CNN) and graph neural networks (GNN), deep learning can achieve high‐precision predictions of drug synergistic or antagonistic effects.^[^ ^99^ ^]^ These computational techniques can systematically identify potential drug combinations in large‐scale heterogeneous data, rigorously predict their clinical effects, thereby significantly accelerating the drug discovery process, while providing innovative research directions for precision medicine and multi‐target drug development. To more clearly illustrate the specific applications and principal technical methods of machine learning and deep learning in drug synergy–antagonism prediction, the following table (**Table** **3**) provides a comprehensive overview of the characteristics, application scenarios, and advantages of different algorithms.

**Table 3:** Applications of machine learning and deep learning methods in synergy–antagonism prediction: comparison of model types, significance, performance metrics, data type, advantages, disadvantages, and related tools.

Method category	Method name	Meaning of the effect	Performance metrics	Data type	Advantages	Limitations	Typical tools/applications
**Machine learning**	Random Forest RF	An ensemble learning method that enhances the accuracy and stability of classification and regression tasks through the combination of multiple decision trees. Applicable in drug discovery, protein–ligand affinity prediction, bioactivity classification, and other fields.	Matthews Correlation Coefficient; Sensitivity (SE);	Molecular Descriptors, etc.	Avoids overfitting; Supports high‐dimensional data; High computational efficiency; Applicable to both classification and regression tasks; Supports variable importance assessment;	Complex model, difficult to interpret; Substantial computational resource requirements; Sensitive to data distribution.	·Wei et al. utilized Random Forest to screen for high A1AR binding affinity antagonists, improving screening efficiency by 90.49%.^[^ ^168^ ^]^ ·Kuz'min et al. developed a new algorithm to explain the contribution of atoms to receptor structure affinity, used for designing novel 5‐HT1A receptor ligands.^[^ ^169^ ^]^
	Decision Tree	A classification and regression model that intuitively represents the decision‐making process through a tree structure. Primarily used in pattern recognition, drug development, bioinformatics, medical diagnostics, and other fields. Through stepwise data partitioning, decision trees can identify key features and provide rules for decision‐making.	Classification Accuracy; Apparent Error Rate; Expected Error Rate, etc.	/	Strong interpretability; Suitable for structurally complex datasets; Can ignore irrelevant variables; Applicable to non‐linear problems.	Prone to overfitting; Excessive tree depth affects generalization capability; Limited effectiveness for high‐dimensional data.	·C4.5^[^ ^170^ ^]^ ·HEAD⁃DT^[^ ^171^ ^]^
	Naïve Bayes NB	A probability‐based classification algorithm based on Bayes' theorem, used to predict the probability of event occurrence; in chemistry and bioinformatics, NB can be used for adverse drug reaction prediction, pharmacophore screening, chemical‐protein interaction modeling, and other applications.	Overall Prediction Accuracy; Matthews Correlation Coefficient; Sensitivity (SE); Specificity (SP), etc.	Molecular Descriptors, etc.	Computationally efficient, suitable for large‐scale data; Applicable to small sample datasets; Does not require feature independence; Suitable for drug discovery tasks.	Assumes complete feature independence; Sensitive to continuous variables; Limited predictive capability compared to deep learning.	B+QSAR pharmacophore modeling successfully screened 10 novel PI3Kα inhibitors, with the best inhibitor showing an IC50 of 0.44 µmol L^−1^, showing promise for anticancer drug development.^[^ ^172^ ^]^
	Support Vector Machine SVM	A classification and regression algorithm based on statistical learning theory that enhances generalization capability by maximizing classification boundaries; performs well on small sample, high‐dimensional variable datasets, suitable for drug discovery, bioactivity prediction, compound toxicity assessment, and other tasks.	Q^2^ (Squared correlation coefficient); RMSEP (Root Mean Square Error of Prediction), etc.	Molecular Descriptors, etc.	Powerful generalization capability; Suitable for high‐dimensional, small sample data; Kernel methods support non‐linear classification; Can be combined with other optimization algorithms; Applicable to various tasks in drug development.	Complex hyperparameter tuning; High computational complexity: Difficult to interpret.	·GA‐CG‐SVM (Genetic Algorithm‐Conjugate Gradient‐SVM) predicts drug‐induced ototoxicity with optimal model prediction accuracies reaching 85.33%–83.05%.^[^ ^173^ ^]^ ·Combining SVM with molecular descriptors, confidence predictors were developed for logD prediction of 91 million compounds.^[^ ^174^ ^]^
	K⁃nearest Neighbor Model KNN	An instance‐based learning algorithm used for classification and regression tasks. It makes predictions by calculating similarities between samples, applicable in fields such as drug property prediction, bioactivity classification, QSAR modeling, and drug–target interaction prediction.	Hamming Loss; AUPR, etc.	Chemical Substructures; Targets, etc.	Simple computation without a training phase; Suitable for high‐dimensional data; Applicable to non‐linear data; Can be combined with other algorithms; Suitable for drug discovery.	Highly susceptible to noise; Sensitive to parameter selection.	·FS‐MLKNN (Feature Selection‐based Multi‐Label KNN) for drug property prediction, improving the accuracy of compound activity prediction.^[^ ^175^ ^]^ ·KNN combined with PaDEL fingerprints for hERG toxicity endpoint prediction, identifying 140 true positive compounds, enhancing the reliability of toxicity predictions.^[^ ^176^ ^]^
**Deep learning**	Convolutional Neural Network CNN	A neural network‐based deep learning model capable of automatically extracting features from high‐dimensional data such as images, molecular structures, and biological sequences, demonstrating excellent performance in drug screening, molecular docking, drug‐disease association prediction, protein‐peptide binding site recognition, and other fields.	AUPR; Precision, etc.	Molecular Descriptors; etc.	Automatic feature extraction; Efficient parameter optimization; Suitable for high‐dimensional data; Can be integrated with other neural networks.	Dependent on large amounts of data; High computational resource requirements; A “black box” model with non‐transparent decision processes that are difficult to interpret.	·CNN‐DDI utilizes convolutional operations of deep learning to extract drug molecular features, combining chemical structure, target information, or molecular fingerprints for drug‐drug interaction prediction.^[^ ^177^ ^]^
	Graph Neural Network GNN	Utilizes graph neural networks to learn complex interaction relationships between drugs and targets, extracting key features of synergistic or antagonistic effects to enhance prediction accuracy.	Balanced Accuracy, etc.	Gene Expression Profiles; Drug Features, etc.	Suitable for modeling complex drug–target relationships; Supports cross‐modal data fusion; Applicable to zero‐shot drug prediction.	High computational complexity; Requires high‐quality graph data.	·DeepDDS^[^ ^178^ ^]^
	Fully Connected Neural Network, FCNN	Processes structured data through a fully connected structure of multiple neuron layers, effectively modeling non‐linear relationships in data and extracting complex patterns from large‐scale datasets. Therefore, FCNN is particularly suitable for tasks involving feature extraction from high‐dimensional data, such as drug molecular structure data or genomic data.	Accuracy; Precision, etc.	Drug Property Features, etc.	High flexibility; Parallel computation suitable for processing large‐scale datasets in bioinformatics; Strong performance with significant drug prediction capabilities.	Excessive data dependency; Prone to overfitting; Requires substantial computational resources, resulting in high computational costs.	·DeepSynergy^[^ ^104^ ^]^ ·MatchMaker^[^ ^147^ ^]^
	Visible Neural Network VNN	A highly interpretable deep learning model used to simulate the hierarchical structure of biological systems, capable of revealing underlying mechanisms of specific processes. VNN integrates prior biological knowledge (such as gene ontology) and has application value in cancer drug response prediction, synergistic drug combination research, and other fields. Strong interpretability; Integration of prior knowledge; Used for synergistic drug combination ranking.	Overall Spearman Correlation Coefficient (rho), etc.	Morgan Molecular Fingerprints, Genotype Representation (binary vector), etc.	Strong interpretability; Integration of prior knowledge; Used for synergistic drug combination ranking.	Prediction accuracy may be lower than other black‐box models; Requires extensive prior knowledge.	· DrugCell^[^ ^107^ ^]^
	Recurrent Neural Network RNN	A deep learning model suitable for sequence data (such as time series, gene sequences, SMILES molecular representations); its recursive structure can capture sequence patterns and chemical bond connectivity relationships in molecular structures, used for drug generation, target prediction, molecular optimization, drug‐disease association analysis, and other tasks.	Natural Product‐Likeness; Reproducibility, etc.	Molecular Structure Data, etc.	Strong capability in processing sequence data; Supports drug generation and molecular optimization; Long Short‐Term Memory (LSTM) and Gated Recurrent Unit (GRU) enhance model performance; Can be used for molecular library optimization and structure generation.	Gradient vanishing and gradient explosion problems; High computational costs; Difficult to accelerate through parallelization.	The quasi‐biomolecular generator QBMG based on GRU‐DNN trained RNN models provides new approaches for precision drug design and bioactive compound screening.^[^ ^179^ ^]^
	Transformer	Demonstrates powerful feature extraction capabilities and multimodal data fusion abilities in synergistic drug combination prediction tasks, capable of learning complex interactions between drug‐gene, drug‐cell line, and gene‐gene relationships, improving the accuracy and generalization capabilities of drug combination predictions.	Cohen's Kappa; Balanced Accuracy; True Positive Rate, etc.	Molecular Structure Features, etc.	Powerful feature extraction capability; Multimodal data fusion; Supports few‐shot learning and zero‐shot learning; Highly scalable and flexible.	High computational resource requirements; Diminishing marginal returns on large datasets; May exhibit hallucination phenomena.	·DTSyn^[^ ^180^ ^]^ ·SynerGPT ·CancerGPT^[^ ^181^ ^]^
	Autoencoder, AE	A neural network model used for data dimensionality reduction, feature extraction, and molecular generation, composed of an Encoder and Decoder that can automatically learn low‐dimensional representations of data, widely applied in drug molecular generation, drug‐protein interaction prediction, drug‐disease association analysis, and other fields.	Accuracy; True Positive Rate, etc.	Molecular Structure Features, etc.	Automatic feature extraction; Data dimensionality reduction; Supports molecular generation and property optimization; Enhances drug‐disease association prediction.	Relatively weak interpretability.	·Gaussian interaction profile kernel and autoencoder, GIPAE^[^ ^182^ ^]^

Abbreviations: HEAD‐DT: Hyper‐heuristic Evolutionary Algorithm for automatically Designing Decision‐Tree algorithms, CNN‐DDI: Convolutional Neural Network Drug‐drug interactions, DeepDDS: Deep Learning for Drug‐Drug Synergy prediction, QBMG: Quasi‐biogenic molecule generator, GRU‐DNN: recurrent neural networks with gate recurrent unit, DTSyn: dual‐transformer‐based neural network, AUPR: Area Under the Precision‐Recall curve.

### 4.2 AI Prediction Models Integrating Multi‐Source Data

#### 4.2.1 Integration of Chemical Structure, Genomics, and Pharmacological Data

AI models that integrate chemical structure, genomics, and pharmacological data establish systematic associations between drug chemical properties, target gene expression, and biological activities,^[^ ^100^, ^101^ ^]^ thereby comprehensively characterizing drug properties. Furthermore, AI techniques are capable of capturing complex interaction patterns of drug combinations at molecular, genetic, and pharmacological levels, which significantly enhances prediction accuracy.^[^ ^24^ ^]^ As a representative example, the canSAR‐2024 database systematically integrates whole genome/exome sequencing (WGS/WES), RNA transcriptome data, chemical and bioactivity information, protein structure data, and clinical trial data, thereby providing comprehensive support for drug target identification and ligandability assessment. The canSAR platform effectively identifies potential drug targets through in‐depth analyses of protein 3D structures. This system combines data from the Protein Data Bank (PDB) and AlphaFold2 prediction models, and applies positive‐unlabeled (PU) learning and random forest algorithms, successfully predicting and validating multiple novel drug‐binding sites and consequently accelerating the drug discovery process.^[^ ^102^ ^]^

#### 4.2.2 Integration of Prior Biological Knowledge

AI models effectively integrate prior biological knowledge, such as signaling pathways, protein–protein interaction (PPI) networks, and metabolic pathways, thereby systematically combining biological context with data‐driven learning.^[^ ^103^ ^]^ This integration strategy significantly enhances the biological plausibility of models by leveraging known biological information to constrain the prediction process,^[^ ^104^ ^]^ thereby preventing results from deviating from established biological mechanisms. The similarity‐based drug response prediction (SDRP) model, which integrates multi‐source data of drugs and cell lines in conjunction with similarity network fusion technology, achieves accurate prediction of IC50 values for cancer drug responses. Compared to existing prediction models, the multi‐source data drug response prediction (MSDRP) model demonstrates significant advantages across evaluation metrics such as root mean square error (RMSE), mean absolute error (MAE), and Pearson correlation coefficient (r), thus substantiating the method's excellent predictive capability and potential clinical applications.^[^ ^105^ ^]^

### 4.3 Current Challenges and Limitations of Artificial Intelligence Methods in Drug Synergy–Antagonism Prediction

Despite revolutionary advances in AI, particularly within machine learning‐based models (such as deep learning), which have achieved significant breakthroughs in predicting drug synergistic and antagonistic effects, their applications continue to face numerous challenges that urgently necessitate a systematic and critical review. Most existing AI models rely on specific structured datasets (e.g., DrugComb, LINCS, GDSC) and consequently struggle to adapt to the complexity and missing data issues inherent in heterogeneous clinical data. The insufficient interpretability of deep learning models also poses a significant limiting factor. Although models such as convolutional neural networks (CNNs), graph neural networks (GNNs), and transformers^[^ ^99^ ^]^ demonstrate superior performance, their “black box” characteristics hinder the theoretical validation of prediction results in biological and clinical practice.^[^ ^106^ ^]^ Prediction results lacking biological context prove difficult to validate experimentally or apply to clinical scenarios. To address this, explainable artificial intelligence methods (e.g., VNN) ^[^ ^107^ ^]^have been proposed in recent years, integrating biological knowledge and improving model transparency to some extent. However, they still face challenges such as the complexity of biological knowledge structures and insufficient generalizability, requiring further enhancement of model flexibility and broad applicability. Most importantly, universal model evaluation metrics or standardized datasets have not yet been established, which makes it difficult to conduct cross‐comparisons of results between different studies. Therefore, future research needs to prioritize the development of unified evaluation frameworks.

## 5 Applications of Drug Synergy–Antagonism in Disease Treatment

### 5.1 Applications of Drug Synergy in Cancer Treatment

#### 5.1.1 Synergy Between Targeted Therapies, Chemoradiotherapy Agents, and Immunotherapy

The combination of multiple drugs holds significant value in cancer treatment.^[^ ^108^ ^]^ Targeted therapies work by interfering with and blocking tumor cell growth and metastasis processes, thereby halting tumor progression;^[^ ^109^, ^110^ ^]^ whereas chemoradiotherapy agents kill tumors by damaging the DNA structure of rapidly proliferating cancer cells.^[^ ^111^, ^112^ ^]^ Simultaneously, immunotherapy activates patients' own immune systems, inducing immune responses to attack cancer cells, while cell therapy primarily combats disease by processing and reinfusing patients' immune cells or stem cells ex vivo, enhancing their antitumor activity.^[^ ^113^, ^114^, ^115^ ^]^ The integration of multiple treatment modalities can form multi‐level, multi‐targeted anticancer networks, thereby enhancing the efficacy against cancer cells and improving overall therapeutic outcomes. In cancer treatment, drug synergy applications are extensive, as demonstrated by several key examples. These include the widespread clinical use of EGFR‐tyrosine kinase inhibitors (EGFR‐TKIs) combined with chemotherapy agents in non‐small cell lung cancer (NSCLC) treatment,^[^ ^116^, ^117^ ^]^ the combination of immunotherapy (anti‐PD‐1 drug Spartalizumab) with targeted therapies (dabrafenib, trametinib) for patients with BRAF‐mutated metastatic colorectal cancer,^[^ ^118^ ^]^ and cytokine‐induced killer (CIK) cell therapy that has shown potential synergistic antitumor efficacy when combined with PD‐1 inhibitors in multiple studies,^[^ ^119^ ^]^ among other research findings.

#### 5.1.2 Multi‐Target Drug Combination Strategies

Multi‐target drug combination strategies effectively inhibit cancer cell proliferation and metastasis by simultaneously targeting multiple signaling pathways or molecular targets.^[^ ^120^ ^]^ Sunitinib, a clinically established targeted therapy for lung cancer, is an oral multi‐target tyrosine kinase inhibitor that specifically inhibits multiple tumor‐related signaling pathways, including vascular endothelial growth factor receptors VEGFR1, VEGFR2, VEGFR3, and FMS‐like tyrosine kinase 3 (FLT3). In the non‐cancer therapeutic field, the investigational compound IHCH‐7179 exhibits properties of a potential multi‐target, multi‐functional drug, demonstrating significant potential for treating various psychiatric disorders and cognitive deficits.^[^ ^75^, ^121^, ^122^ ^]^ The strategic application of multi‐target immune checkpoint inhibitors has emerged as a critical research focus for improving the efficacy of tumor immunotherapy. By combining inhibitors targeting different immune checkpoints, such as the dual blockade strategy of cytotoxic T‐lymphocyte‐associated antigen 4 (CTLA‐4) and programmed death receptor 1 (PD‐1), regulatory approval has been secured for treating various advanced malignancies, yielding significant therapeutic benefits in clinical practice. Additionally, combined inhibition strategies targeting next‐generation immune checkpoint molecules—such as T‐cell immunoglobulin and mucin domain‐3 (TIM‐3), lymphocyte activation gene‐3 (LAG‐3), and T‐cell immunoreceptor with immunoglobulin and ITIM domain (TIGIT)—are currently undergoing clinical trials, aiming to overcome the resistance mechanisms and efficacy limitations associated with single‐target immunotherapy.^[^ ^123^, ^124^ ^]^

### 5.2 Drug Synergy in Infectious Disease Treatment

#### 5.2.1 Synergistic Effects of Antimicrobial Agents

The synergistic effect of antimicrobial agents refers to the simultaneous use of two or more antimicrobial drugs, where the antibacterial effect is significantly greater than the sum of effects when each drug is used alone.^[^ ^125^ ^]^ Recent studies^[^ ^126^ ^]^ have established that phage‐antibiotic synergy (PAS) represents a potentially effective strategy for treating multidrug‐resistant pathogens. This approach not only significantly reduces bacterial load but also slows the development of bacterial resistance to both phages and antibiotics, thereby achieving more potent inhibition of pathogens.^[^ ^127^ ^]^ When β‐lactam antibiotics are synergistically applied with aminoglycosides, the former disrupt bacterial cell wall synthesis, facilitating the penetration of aminoglycosides into the bacterial inner membrane, thereby enhancing the overall antimicrobial efficacy. Current research demonstrates that combinations of sulbactam with cefuroxime (CXM), or sulbactam with CXM accompanied by β‐lactamase inhibitors (such as avibactam or durlobactam), consistently exhibit significant synergistic effects. The synergistic mechanism of different β‐lactam drug combinations in inhibiting L, D‐transpeptidase—an enzyme critical for major peptidoglycan cross‐linking reactions in Mycobacterium abscessus (Mab)—represents an important current research area. These combination drug strategies have been successfully applied to treat macrolide‐resistant Mab infections.^[^ ^128^ ^]^

#### 5.2.2 Synergistic Effects of Antiparasitic Drugs

The synergistic effect of antiparasitic drugs refers to the strategic combined application of two or more antiparasitic agents, which aims to enhance therapeutic outcomes and overcome the limitations associated with single‐drug treatment.^[^ ^129^ ^]^ This synergistic effect is primarily achieved by simultaneously interfering with multiple key biological pathways of parasites. A well‐documented example involves the combined application of artemisinin compounds with other antimalarial drugs, which not only significantly enhances antimalarial efficacy but also effectively delays the development of parasite resistance.^[^ ^130^, ^131^ ^]^ Antiparasitic drug combination strategies based on synergistic mechanisms have demonstrated significant clinical value in treating complex parasitic infections such as malaria and leishmaniasis, thereby not only improving treatment efficiency but also reducing treatment failure rates and mitigating adverse reactions.^[^ ^132^ ^]^

### 5.3 Drug Synergy in Metabolic Disease Treatment

#### 5.3.1 Synergistic Effects of Hypoglycemic Agents

The synergistic mechanism of hypoglycemic agents refers to the achievement of more effective blood glucose control through the combined use of multiple glucose‐lowering drugs, wherein different types of hypoglycemic agents act on multiple metabolic processes such as insulin secretion, glucose absorption, and hepatic glucose output, thereby producing synergistic effects.^[^ ^133^ ^]^ For example, when metformin is combined with SGLT2 inhibitors, metformin primarily lowers blood glucose by inhibiting hepatic glucose production and improving insulin sensitivity, while SGLT2 inhibitors (empagliflozin and dapagliflozin) lower blood glucose by inhibiting sodium‐glucose cotransporter 2 in the kidneys, thereby promoting the excretion of excess glucose in the urine.^[^ ^134^, ^135^ ^]^ The former reduces glucose production, while the latter increases glucose excretion; consequently, these two mechanisms complement each other to achieve comprehensive blood glucose level control. This multi‐target synergistic strategy not only significantly enhances blood glucose management in diabetic patients but also minimizes potential side effects associated with monotherapy.

#### 5.3.2 Synergistic Effects of Lipid‐Lowering Agents

The synergistic mechanism of lipid‐lowering agents primarily operates by effectively reducing serum cholesterol and triglyceride levels through the combined use of multiple lipid‐lowering drugs, wherein different types of lipid‐lowering agents regulate lipid metabolism processes through their unique pathways, thereby producing significant synergistic effects.^[^ ^136^ ^]^ For example, statins significantly inhibit de novo cholesterol synthesis by targeting 3‐hydroxy‐3‐methylglutaryl coenzyme A (HMG‐CoA) reductase,^[^ ^137^ ^]^ while fenofibrate, as a selective peroxisome proliferator‐activated receptor α (PPARα) agonist, not only possesses multiple functions in regulating lipid metabolism but also exhibits non‐lipid regulatory effects; their combined application acts synergistically to more effectively reduce serum total cholesterol and low‐density lipoprotein cholesterol (LDL‐C) levels. This combination therapy strategy based on multi‐target synergistic mechanisms is particularly significant for high‐risk cardiovascular disease patients, not only offering more comprehensive and effective control of dyslipidemia but also significantly reducing the incidence of major adverse cardiovascular events (MACE) and associated mortality risks.

## 6 Future Perspectives

**Figure 3:**
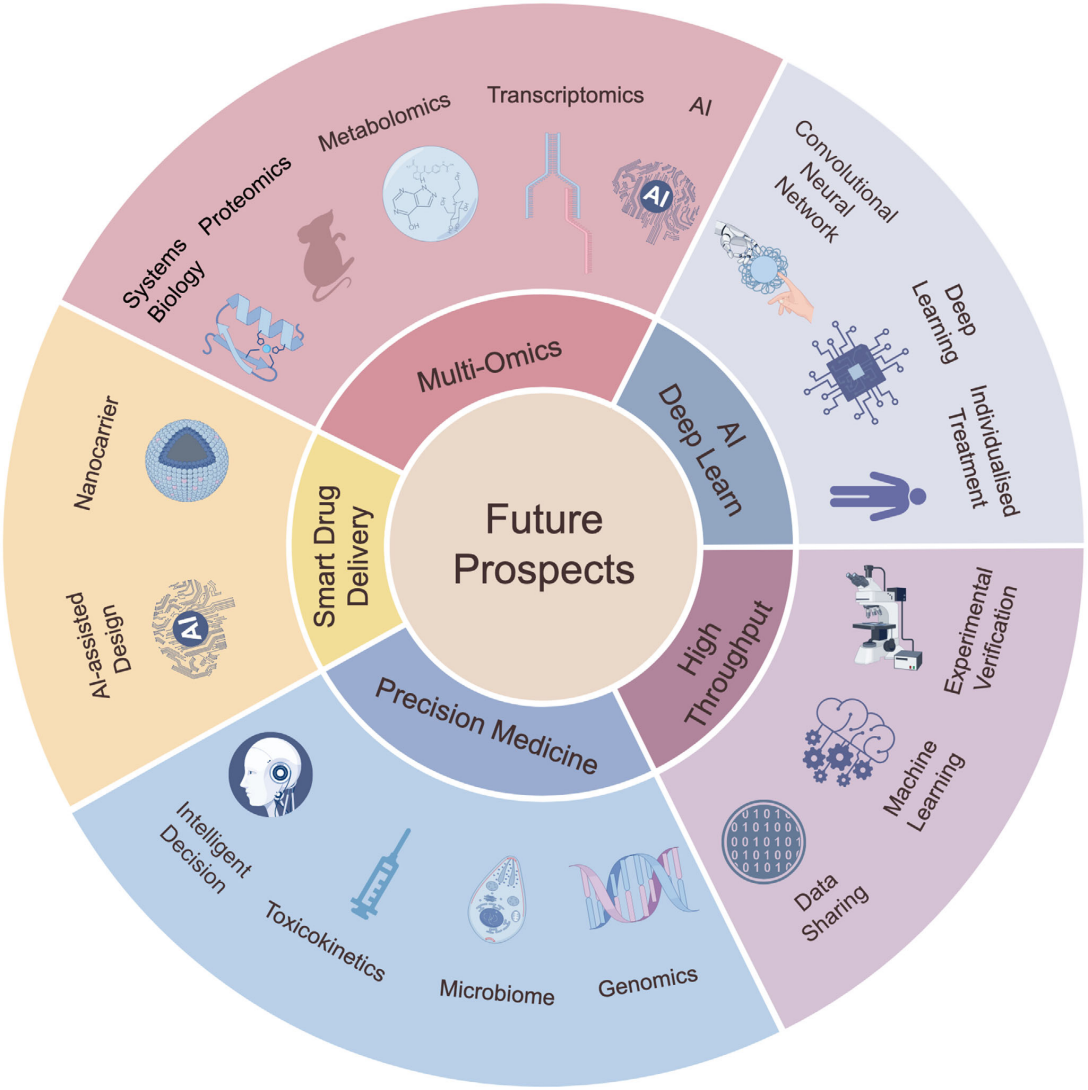
Five critical future development directions in drug synergy–antagonism research. This figure highlights the potential applications of these five key directions in future medical advancements: 1) Multi‐omics data integration: proteomics, systems biology, metabolomics, transcriptomics, and artificial intelligence (AI) deep analysis algorithms. 2) Intelligent drug delivery systems: nanocarrier technology and AI‐assisted therapeutic decision‐making. 3) Precision medicine: genomics, precise toxicity regulation, intelligent treatment decisions, and microbiome analysis. 4) High‐throughput experimental techniques: initial screening with machine learning (ML) algorithms, large‐scale data sharing, and systematic experimental validation. 5) AI‐assisted drug development: deep learning models, convolutional neural network architectures, and targeted personalized treatment strategies. This figure was created using visualization tools provided by the Figdraw platform.

Future research on drug synergy–antagonism will rely on the integrated development of cutting‐edge technologies, including multi‐omics data integration, high‐throughput experimental techniques, precision medicine, intelligent drug delivery systems, and artificial intelligence (**Figure** **3**). The integration of multi‐omics data will comprehensively describe the effects of drugs on cellular pathways and metabolic networks,^[^ ^138^ ^]^ while advancements in artificial intelligence (AI), especially deep learning, will enable personalized drug synergy predictions, thereby improving the predictive capabilities of combination therapies. The combination of high‐throughput experiments and computational methods will significantly improve the efficiency of drug combination screening and optimize model prediction capabilities through rigorous experimental validation. In precision medicine, genomics and microbiomics will exert a greater impact on drug combination decisions, ultimately leading to the development of more tailored personalized treatment plans. Additionally, novel drug delivery systems (such as intelligent nanocarriers) will enhance drug synergistic effects by precisely controlling drug release, minimizing antagonistic effects, and promoting targeted therapeutic outcomes. The integration of these technologies will significantly improve the clinical efficacy of combination therapies, accelerate the development of precision medicine, and provide more effective and safer therapeutic approaches for treating complex diseases in the future.

### 6.1 Integration of Multi‐Omics Data in Drug Synergy–Antagonism Research

Proteomics elucidates the relationship between drugs and protein expression and modification,^[^ ^139^ ^]^ metabolomics provides comprehensive information on how drugs affect metabolites,^[^ ^140^ ^]^ while transcriptomics demonstrates the regulatory effects of drugs on gene expression.^[^ ^141^ ^]^ By integrating multi‐omics data, researchers can further understand the overall impact of drugs on cellular pathways and metabolic networks, thereby comprehensively elucidating potential synergistic or antagonistic mechanisms between drugs. Systems biology employs a holistic and integrated approach, which is distinct from reductionist methods, enabling researchers to more comprehensively understand complex biological processes. The application of systems biology and multi‐omics data integration has proven crucial for studying drug synergistic and antagonistic effects. To achieve a more comprehensive analysis of the complex biological processes involved in drug interactions, proteomics, metabolomics, and transcriptomics data can be effectively combined with sophisticated computational tools and artificial intelligence algorithms.^[^ ^142^ ^]^

### 6.2 Application of Artificial Intelligence Technologies in Drug Synergy–Antagonism Research

Simulation plays a key role in drug synergy–antagonism research; in the coming decades, artificial intelligence, especially deep learning, will facilitate more accurate, personalized, and universal drug synergy predictions, thus enabling tailored therapeutic regimens for patients, as “one‐size‐fits‐all” approaches increasingly fail to meet the growing demands for drug synergy solutions. Complex computational methods such as message‐passing models, networks that preserve spatial symmetry, and hybrid de novo designs can significantly optimize predictions of drug combinations, thereby enhancing the accuracy in determining synergistic and antagonistic effects. Advances in open data sharing and model development are expected to help accurately identify the most appropriate drug combinations in personalized medicine, minimize exposure to drugs with adverse effects, and improve therapeutic outcomes.^[^ ^143^ ^]^ Future challenges include enhancing the ability to accurately predict the efficacy and safety of drug candidate molecules and improving the interpretability of AI models (such as those utilizing convolutional neural networks) to decipher complex interactions between drugs, targets, and diseases.^[^ ^144^ ^]^ PIDB and related tools are poised to lay the foundation for these exciting advances, as evolving AI technologies integrated with structural biology will drive optimal drug synergy–antagonism screening strategies, and expedite the implementation of precision treatment plans.

### 6.3 Applications of High‐throughput Experimental Technologies Combined with Computational Methods in Drug Synergy–Antagonism Research

In contrast to traditional experimental methods that can only identify a limited number of drug combination effects, the utilization of mathematical and statistical models through response surface modeling to evaluate combined drug actions can significantly expand this search space.^[^ ^23^, ^101^, ^103^ ^]^ Large‐scale biomedical databases and machine learning methods, especially deep learning, demonstrate significant application potential in developing computational prediction models for drug synergy.^[^ ^145^ ^]^ However, the basic reasoning and decision‐making processes of current artificial intelligence models are often extremely complex and difficult to understand, thereby lacking sufficient transparency. Moving forward, by integrating models with experimental data and computational predictions, researchers can potentially establish more accurate drug synergy–antagonism prediction systems. Traditional in vitro experimental methods are typically time‐consuming, labor‐intensive, and resource‐demanding. In contrast, when machine learning (ML) is used to predict drug synergy, it can systematically screen potential synergistic drug combinations to provide a solid scientific basis for subsequent experimental validation, effectively overcoming these limitations of traditional experiments and significantly improving research efficiency and cost‐effectiveness. Next‐generation high‐throughput technologies will expand both the breadth and depth of data collection, thereby providing a more reliable foundation for accurately predicting the effects of drug combinations. Future development will focus on optimizing computational models to improve the accuracy of synergistic and antagonistic effect predictions, and through rigorous experimental validation, aim to achieve intelligent optimization of drug combination strategies, ultimately promoting clinical applications of personalized therapy.

### 6.4 Advances in Drug Synergy–Antagonism Research in the Context of Precision Medicine

The primary aim of personalized medicine is to select the most suitable synergistic or antagonistic drug combinations for patients based on their unique characteristics, such as genomic composition and drug response, thereby enabling clinicians to adjust treatment strategies, enhance therapeutic effects, and reduce adverse reactions.^[^ ^146^ ^]^ Further research based on genomic and phenotypic data predictions significantly enhances the application of personalized medicine in drug therapy, resulting in treatment regimens that are more precise and effective. The development of intelligent decision support systems that leverage artificial intelligence technologies such as deep learning frameworks and machine learning algorithms to predict drug synergy scores provides clinicians with optimized drug combination recommendations for formulating more precise personalized treatment plans.^[^ ^147^ ^]^ Numerous studies have demonstrated that the microbiome exerts regulatory effects on the bioavailability, biotransformation, distribution, excretion, and potential toxicity of various drugs. These drugs include antibiotics, antihypertensives, antidiabetic agents, anticancer drugs, antipsychotics, and analgesics.^[^ ^148^ ^]^ However, the relationship between drug combination effects and the microbiome remains a complex area that requires further in‐depth investigation. In the case of combination therapy, interactions between different drugs are not only influenced by the drugs themselves but are also regulated by microbial communities. Antibiotics can alter the diversity and function of gut microbiota, which may affect the metabolism of other drugs.^[^ ^149^, ^150^ ^]^ Particularly when antimicrobial therapy is combined with other drugs (such as antidiabetic or antipsychotic medications), changes in the microbiota can lead to decreased drug efficacy or increased toxicity.^[^ ^151^, ^152^, ^153^, ^154^, ^155^ ^]^ For anticancer drugs, gut microbiota can influence the effectiveness of cancer treatment by altering immune responses and drug metabolism, potentially enhancing drug effects or reducing toxicity. Therefore, the relationship between drug combination effects and the microbiome requires further research and discussion, an area that holds profound implications for optimizing personalized medicine.

### 6.5 Innovative Applications and Prospects of Novel Drug Delivery Systems Combined with Synergistic–Antagonistic Effects

Recent advances in nanotechnology have overcome clinical challenges faced by traditional drug delivery, such as severe systemic toxicity, low bioavailability, and drug resistance in target cells.^[^ ^156^ ^]^ Future intelligent nanocarrier designs will achieve the ability to simultaneously deliver multiple drugs and, through precisely controlled release systems, ensure that drugs produce synergistic effects at optimal times and doses, thereby significantly enhancing therapeutic outcomes. The development of targeted delivery systems aims to precisely transport drugs to target tissues or specific cells, thereby increasing drug concentration in localized areas and enhancing synergistic effects between drug molecules.^[^ ^157^ ^]^ These systems can reduce drug distribution in non‐target tissues through their high targeting specificity, thus effectively lowering systemic side effects and significantly improving treatment safety and efficacy. With advanced artificial intelligence technologies, researchers can analyze massive datasets to precisely predict drug behaviors and systematically simulate drug formulations and delivery processes, thereby gaining deep insights into the molecular mechanisms of drug delivery. This technological advancement enables researchers to systematically evaluate multiple complex scenarios, efficiently optimize delivery systems, and significantly reduce both experimental iterations and resource consumption. By integrating the aforementioned strategies, researchers can scientifically optimize drug delivery system designs, precisely select optimal drug combinations and delivery parameters, and maximize synergistic effects while minimizing antagonistic actions, thereby significantly improving therapeutic outcomes and patient prognosis.^[^ ^158^ ^]^

## Conflict of Interest

The authors declare no conflict of interest.

## Author Contributions

A.L., C.C., A.J., and C.Q. contributed equally to this work and share first authorship. A.Q.L, C.C., A.M.J., C.Q., A.G., Z.J.Z., Z.R.Z., Z.Q.L., Z.Y.Z., Q.C., S.F.Y., and P.L. wrote the original draft; Q.C., S.F.Y. and P.L. conceptualized the study; A.Q.L, C.C., A.M.J., Z.Q.L., Z.Y.Z., and C.Q. performed the investigations; A.Q.L, C.C., A.M.J., C.Q., A.G., Z.J.Z., Z.R.Z., Z.Q.L., Z.Y.Z., Q.C., S.F.Y., and P.L. reviewed and edited the final manuscript; A.Q.L., Q.C., S.F.Y., and P.L. supervised the study. All authors have read and agreed to the published version of the manuscript.
